# State variation in effects of state social distancing policies on COVID-19 cases

**DOI:** 10.1186/s12889-021-11236-3

**Published:** 2021-06-28

**Authors:** Brystana G. Kaufman, Rebecca Whitaker, Nirosha Mahendraratnam, Sophie Hurewitz, Jeremy Yi, Valerie A. Smith, Mark McClellan

**Affiliations:** 1 Margolis Center for Health Policy, Duke University, 230 Science Drive, Durham, NC 27705 USA; 2 Population Health Sciences, Duke University School of Medicine, Durham, NC USA; 3 Center of Innovation to Accelerate Discovery and Practice Transformation (ADAPT), Durham VA Medical Center, Durham, NC USA; 4 General Internal Medicine, Duke University School of Medicine, Durham, NC USA

**Keywords:** COVID-19, Social distancing, Public health, Health policy, Outcomes research

## Abstract

**Background:**

The novel coronavirus disease 2019 (COVID-19) sickened over 20 million residents in the United States (US) by January 2021. Our objective was to describe state variation in the effect of initial social distancing policies and non-essential business (NEB) closure on infection rates early in 2020.

**Methods:**

We used an interrupted time series study design to estimate the total effect of all state social distancing orders, including NEB closure, shelter-in-place, and stay-at-home orders, on cumulative COVID-19 cases for each state. Data included the daily number of COVID-19 cases and deaths for all 50 states and Washington, DC from the New York Times database (January 21 to May 7, 2020). We predicted cumulative daily cases and deaths using a generalized linear model with a negative binomial distribution and a log link for two models.

**Results:**

Social distancing was associated with a 15.4% daily reduction (Relative Risk = 0.846; Confidence Interval [CI] = 0.832, 0.859) in COVID-19 cases. After 3 weeks, social distancing prevented nearly 33 million cases nationwide, with about half (16.5 million) of those prevented cases among residents of the Mid-Atlantic census division (New York, New Jersey, Pennsylvania). Eleven states prevented more than 10,000 cases per 100,000 residents within 3 weeks.

**Conclusions:**

The effect of social distancing on the infection rate of COVID-19 in the US varied substantially across states, and effects were largest in states with highest community spread.

**Supplementary Information:**

The online version contains supplementary material available at 10.1186/s12889-021-11236-3.

## Background

The severe acute respiratory syndrome coronavirus 2 (SARS-CoV-2) caused the coronavirus disease 2019 (COVID-19) pandemic, which sickened over 20 million residents and caused over 370,000 deaths in the United States (US) by January 2021 [1, 2]. This highly contagious, novel disease has a high case fatality rate in high-risk populations, and can cause severe morbidity and high healthcare resource use. In the US, each state implemented a combination of social distancing policies in order to mitigate transmission, including limiting the size of gatherings and closing schools and non-essential businesses [3]. States’ implementation strategies varied, yet little is known about how the impact of social distancing policies varied between states.

In the absence of therapeutics and contact tracing capacity early on in the pandemic, COVID-19 mitigation and suppression strategies in the US relied on social distancing policies to prevent the spread of the disease and reduce COVID-19-related morbidity and mortality [3]. A variety of social distance interventions were implemented in the US to mitigate the spread of COVID-19, including limits on the size of group gatherings, public schools and non-essential business (NEB) closure, and shelter in place or stay at home orders [4]. These social distancing and mitigation policies changed behavior, specifically reduced mobility and gathering, which reduced the opportunities for the virus to spread from person to person. Early in the pandemic, simulation models suggested that social distancing policies can provide crucial time to increase healthcare capacity [5]. The effectiveness of social distancing orders in mitigation community transmission has been well established [4, 6, 7].

States varied in their timing, sequencing, implementation and enforcement for social distancing policies. The Centers for Disease Control and Prevention (CDC) provided guidance on responding to COVID-19; however, state and local governments were responsible for implementing social distancing policies. Furthermore, variation in timing of policies can impact the effectiveness, with earlier intervention delaying epidemic curve and later intervention flattening the epidemic curve [5, 8, 9]. Thus, the variation in states’ implementation strategies may result in variation in the effect of social distancing measures between states.

The effectiveness of social distancing orders in mitigating community transmission has been well established; however, little is known about the variation in the effect across states.

The objective of this study was to describe state variation in the effect of initial social distancing policies and non-essential business (NEB) closure on infection rates early in 2020. This study expands knowledge by estimating the impact for each state using state-specific trends in spread of COVID-19.

## Methods

We used an interrupted time series (ITS) quasi-experimental study design to evaluate the total effects of social distancing policies implemented between January 21 and May 7, 2020, using non-essential business (NEB) and public school closures as the key implementation markers [10]. The study period ends May 7, 2020 to prevent reopening of NEB from impacting the estimates of closure [7]. The ITS design compares COVID-19 rates among states that implemented NEB closure to the predicted rates assuming the trend in cases would have been consistent in the absence of the intervention. This assumption allows us to estimate the effect of social distancing across all states as the average difference between the predicted case rate with closure and the predicted case rate without closure.

### Data and sample

Dates of social distancing orders were obtained from the Boston University School of Public Health COVID-19 US State Policy Database from January 21, 2020 to May 7, 2020 [10]. Our sample included 50 U.S. states and the District of Columbia. We excluded US territories due to limited data on mitigation policies. Daily COVID-19 confirmed case counts were obtained from the New York Times state-level database [11]. State population data were obtained from the 2018 American Community Survey [12].

The primary outcome was the daily cumulative number of COVID-19 cases in the state from January 21, 2020 to May 7, 2020. The outcome was lagged by 5 days because the effect of the intervention on confirmed cases was expected to be delayed by the incubation period as well as time for reporting. A shorter lag time was preferred to avoid classifying daily outcomes from the post-period in the pre-period, and prior evidence suggested that effects are significant as early as 5 days after implementation [4]. The secondary outcome was the number of confirmed COVID-19 deaths, lagged by 10 days.

We estimated the combined effect of two social distancing measures: state-level NEB and public school closures. We defined the start of the intervention as the date of public school closure, and we estimated the change in slope between school closure and NEB closure (“ramp up” period) as well as the change in slope after NEB closure (“post” period) (Supplemental Material). The total effect of closures included both the change during the ramp up period as well as the change in the post period. A shelter-in-place order included the closure of schools and NEBs, so we used the date of shelter-in-place to define NEB closure if a date was not reported for NEBs specifically.

### Statistical analysis

We predicted cumulative daily cases and deaths using a generalized linear model with a negative binomial distribution and a log link for two models. The model examined the total effect of states’ social distancing orders (including NEB and public school closure). The time variable (day) was centered around the date of school closure, and we truncated observations at 20 days prior to school closure to reduce bias due to testing limitations and periods of zero growth early in the epidemic, and improve estimates of states’ pre-period trends. The mean number of observations per state during the study period was 66.4 (SD = 14), with a mean of 16.5 days prior to the school closure and 26.5 days prior to NEB closure (Table 1). Thus, the mean number of time points in the post-closure period is > 30, well over the 3–12 time points recommended for interrupted time series analysis [13, 14]. This model included interactions between the treatment variable (categorical; pre-period, ramp-up period, and post-period) and a linear and quadratic time variable to allow the slopes to differ in the ramp-up and post-period compared to the pre-period. Thus, the treatment effect was estimated as the average of the differences in the slope for each state before (pre-period) and after closure.

**Table 1:** Timing of states social distancing policies relative to COVID-19 burden

	Total (***N*** = 51)
	*Mean (SD)*
Days in sample	66.4 (14.0)
Days from index case to school closure	16.5 (14.7)
Cases at school closure	189.8 (391.3)
Deaths at school closure	3.3 (9.0)
Days from index case to NEB closure	26.5 (13.8)
Cases at NEB Closure	1778.6 (2771.1)
Deaths at NEB closure	30.5 (43.7)

*NEB* non-essential businesses, *SD* standard deviation;

We used the method of recycled predictions [15] to compare predicted cumulative cases with social distancing compared to without social distancing at 3 weeks after the date of school closure for each state. In nonlinear models, the method of recycled predictions is recommended for estimating marginal effects because the marginal effect can vary depending on the covariate values. In this method, predictions are estimated for the treated scenario and the non-treated scenario for each observation in the sample holding all other variables constant [16].

State fixed effects controlled for state characteristics associated with outcomes that did not change over the study period. To allow time trends in each state to vary prior to the intervention, we used a unique time trend for each state in all models. We used an offset to account for varying population sizes and present marginal effects as a rate per 100,000 residents. We used robust standard errors to account for heteroskedasticity in measurement of cases over time and clustered standard errors by day to address temporal clustering of outcomes. To improve model fit, we tested higher order terms and alternative distribution assumptions (see statistical supplement). The final model included a quadratic time trend to allow the slope to vary over time. In a sensitivity analysis, we ran models with and without New York and New Jersey included because of the large case counts. All analyses were conducted using Stata 16.0.

## Results

By the end of the study period, all 50 states and the District of Columbia had closed public schools, and all but five states (Arkansas, Nebraska, North Dakota, South Dakota, Wyoming) had NEBs (with or without shelter-in-place, stay-at-home, or similar order). Five states that mandated NEB closure did not order residents to shelter in place (Kentucky, Oklahoma, Texas, Iowa, and Connecticut). Most states (31) closed schools the week between March 13, 2020 and March 20, 2020 while Nebraska closed schools last, on April 3, 2020 (Table 1). While three states closed schools and businesses on the same day (California, Iowa, Maine), most closed schools prior to NEB closure (mean = 9.7 days). States had an average of 189.8 COVID-19 cases and 3.3 related deaths on the date of school closure. States had an average of 1778.6 COVID-19 cases and 30.5 related deaths on the date of NEB closure.

### Effects of social distancing orders on COVID-19 cases and deaths

**Fig. 1:**
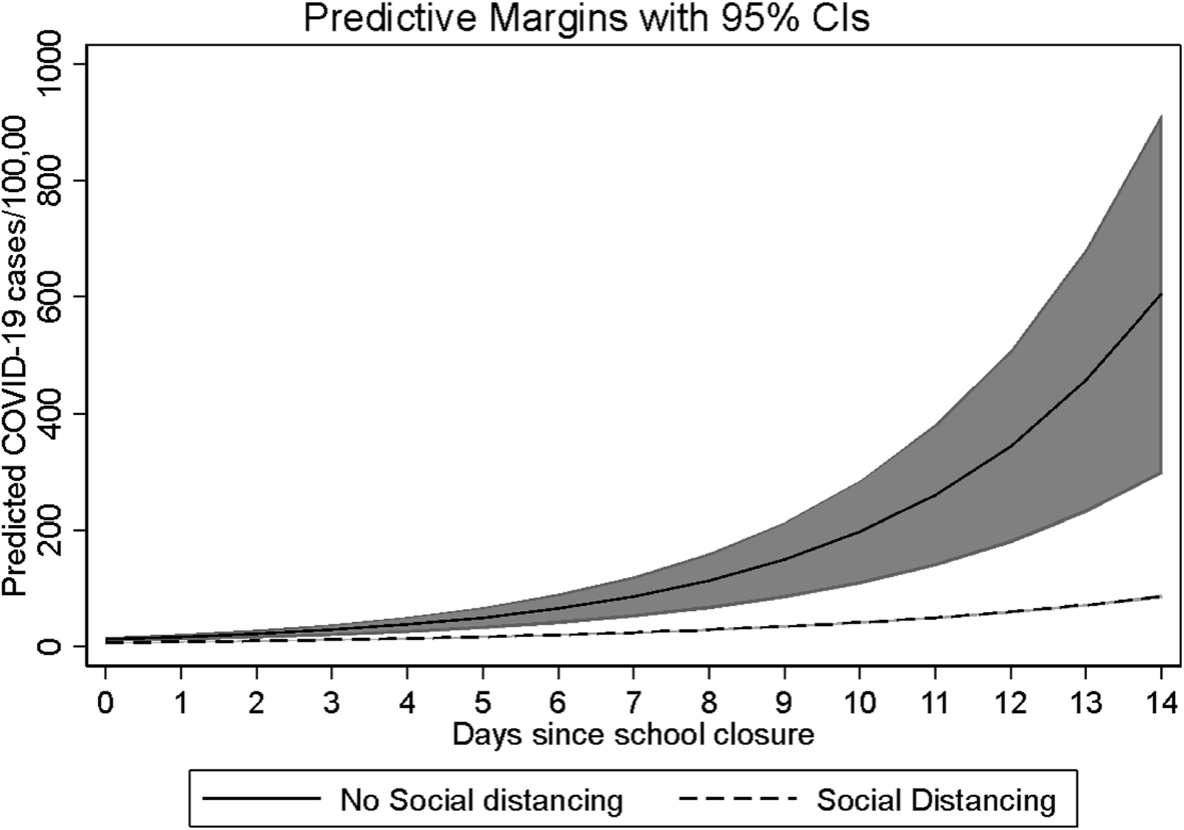
Cumulative incidence of COVID-19 cases with and without state social distancing policies

Social distancing, including closure of NEB and public schools, reduced the daily COVID-19 cases by 15.4% (Relative Risk [RR] = 0.846; Confidence Interval [CI] = 0.832, 0.859) (Table 2). The effect of the relative reduction on cumulative cases per 100,000 increases exponentially over time due to the exponential spread of infection in the absence of the intervention (Fig. 1). The average reduction in cases due to social distancing policies at 2 weeks was − 518.9 (CI = -827.7, − 210.1) cases and at 3 weeks was − 4175 (CI = -6910.1, − 1440.9) per 100,000 residents. Social distancing was associated with a daily reduction in COVID-19 deaths of 11.6% (RR = 0.884; CI = 0.861, 0.906) (Table 2). Predictions for deaths were not precise enough to estimate the number of deaths prevented. Results in sensitivity analyses were similar (Table 2).

**Table 2:** Total effects of social distancing policies on COVID-19 cases and deaths

	Cumulative Daily Cases	Cumulative Daily Deaths
**Total Effects of Social Distancing Policies**	*RR (95% CI)*	*RR (95% CI)*
Primary Analysis (all states)	0.85 (0.83, 0.86)	0.88 (0.86, 0.91)
Sensitivity Analysis ^a^	0.86 (0.85, 0.87)	0.90 (0.87, 0.92)
	*Change in predicted cases per 100,000 (95% CI)*	*Change in predicted deaths per 100,000 (95% CI)*
Difference at 14 days	− 518.92 (− 827.70, − 210.14)	−11.02 (− 23.57, 1.54)
Difference at 21 days	−4175.46 (− 6910.06, − 1440.86)	−77.60 (− 168.81, 13.61)

*RR* relative risk, *CI* confidence interval, *NEB* non-essential businesses

Note: negative numbers represent cases or deaths prevented

^a^ Sensitivity Analysis included all states except New York and New Jersey

**Fig. 2:**
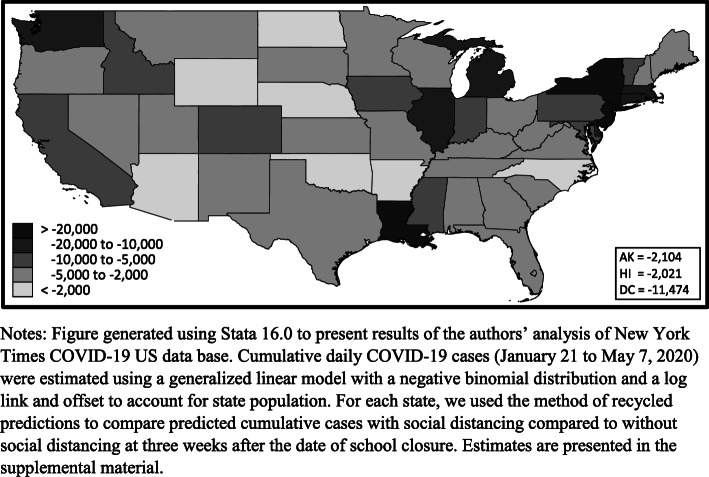
Estimated COVID-19 cases per 100,000 residents prevented by social distancing policies within 3 weeks. Notes: Figure generated using Stata 16.0 to present results of the authors’ analysis of New York Times COVID-19 US data base. Cumulative daily COVID-19 cases (January 21 to May 7, 2020) were estimated using a generalized linear model with a negative binomial distribution and a log link and offset to account for state population. For each state, we used the method of recycled predictions to compare predicted cumulative cases with social distancing compared to without social distancing at 3 weeks after the date of school closure. Estimates are presented in the supplemental material

The impact of social distancing policies varied across states. Eleven states were estimated to have prevented more than 10,000 cases per 100,000 residents including New York, New Jersey, Louisiana, Massachusetts, Connecticut, Michigan, Rhode Island, District of Columbia, Washington, Illinois, and Delaware (Fig. 2). Seven states were estimated to have prevented less than 2000 cases per 100,000 residents including Oklahoma, Arizona, North Dakota, Nebraska, Wyoming, Arkansas, and North Carolina. The Mid-Atlantic census division accounted for half of the nationally prevented cases at 16.5 million cases. State specific estimates are presented in the supplemental material (Table S1).

## Discussion

The effect of state social distancing orders on the infection rate of COVID-19 in the US varied substantially across states. In our study, the effect of social distancing early in the pandemic (March – May 2020) ranged from less than 2000 cases per 100,000 residents in to over 10,000 prevented cases per 100,000 residents. About half (16.5 million) of prevented cases nationally were estimated among residents of the Mid-Atlantic census division (New York, New Jersey, Pennsylvania) where community spread was higher than in other regions of the country at the time.

Our findings confirm prior estimates of the effects of social distancing mandates on the spread of COVID-19 early in the COVID-19 epidemic and expands understanding by describing variation in the effects across states. The relative daily reductions in COVID-19 cases (15.4%) and deaths (11.6%) associated with social distancing orders prevented an estimated 33 million cases nationwide. Similar to our results, prior evidence evaluating the effectiveness of social distancing policies estimated 34 million prevented by the end of April and up to 60 million cases in total [4, 6, 17]. Another study evaluating the timing of school closure found closing schools when the cumulative incidence of COVID-19 was in the lowest quartile compared with the highest quartile was associated with 128.7 fewer cases per 100,000 population over 26 days and with 1.5 fewer deaths per 100,000 population over 16 days [6].

COVID-19 infection rates have continued to increase nationally, and some states and countries are implementing additional rounds of stay at home orders. While significant advances in vaccines mitigate COVID-19 susceptibility, new evidence suggests that the benefits of continuing non-pharmaceutical interventions including social distancing policies is greater than vaccination alone [18]. Similar to other studies, we found the largest effects of social distancing orders were observed in states with high community spread at the time of implementation [6]. Due to the growth in infection rates, states that reinstate stay at home orders may prevent a greater number of COVID-19 cases than were prevented by implementation early in the pandemic, particularly in rural states where the rate of infection in the community was low early in 2020. Emerging coronavirus variants that are more contagious or morbid may cause surges that require physical mitigation measures including social distancing mandates and face masks to reduce transmission [19]. Despite efforts to suppress and contain the virus, COVID-19 is likely to become endemic, particularly among populations with high poverty rates [20].

Measurement error in the number of cases and deaths is a concern as the procedures for testing and documenting cases and deaths have continued to adapt over time and vary by state. Asymptomatic cases and out-of-hospital deaths are most likely to be underrepresented. Our data included COVID-19 cases confirmed by testing, and many cases and deaths may go undetected due to both testing limitations and asymptomatic carriers. In addition, the state level data do not capture the variation in local policies (e.g., the introduction of masking), which may have contributed to the effectiveness of social distancing mandates. States adopted and adapted multiple interventions during the period, and our analysis cannot isolate effects of individual policies, only total effects. This analysis also could not distinguish whether the observed relationships are due to voluntary versus mandated social distancing. Finally, the ability to control for the state-specific trend in mortality was limited due to the few deaths that occurred prior to school closure.

## Conclusions

The effect of social distancing on the infection rate of COVID-19 in the US varied substantially across states and effects were largest in states with highest community spread. The level of community spread should be a key factor for states considering mandating the closure of non-essential businesses and stay-at-home orders.

## Supplementary Information

**Additional file 1: Figure S1.** Comparing Predicted and Observed COVID-19 Cases (lagged 5 days) for Models A-F. **Table S1.** State-specific estimates of COVID-19 cases averted with social distancing policies compared to projected cases without social distancing (3 weeks after school closure).

## Data Availability

The New York Times. (2020). Coronavirus (Covid-19) Data in the United States accessed May 10, 2020 from https://github.com/nytimes/covid-19-data. Boston University School of Public Health COVID-19 US State Policy Database accessed May 10, 2020 from https://github.com/USCOVIDpolicy/COVID-19-US-State-Policy-Database. United States Census American Community Survey accessed May 10, 2020 from https://www.census.gov/programs-surveys/acs/data.html. Code availability (software application or custom code).

## Abbreviations

CI: Confidence interval
ITS: Interrupted time series
NEB: Non-essential business
RR: Relative risk
SARS-CoV-2: Severe acute respiratory syndrome coronavirus 2
US: United States
