# Supplementary material for: State variation in effects of state social distancing policies on COVID-19 cases
Source: BMC Public Health. 2021 Jun 28;21:1239. doi: 10.1186/s12889-021-11236-3 (PMC8237534; doi:10.1186/s12889-021-11236-3)
Supplement: Supplementary file 1 — Additional file 1: Figure S1. Comparing Predicted and Observed COVID-19 Cases (lagged 5 days) for Models A-F. Table S1. State-specific estimates of COVID-19 cases averted with social distancing policies compared to projected cases without social distancing (3 weeks after school closure). [file 12889_2021_11236_MOESM1_ESM.docx]

**D**. Negative Binomial

AIC=16.4

AIC=16.4

AIC=16.8

**E**. Gamma

COVID-19 Cases (log)

COVID-19 Cases (log)

COVID-19 Cases (log)

**C**. Cubic Pre-Trend

Predicted Cases (log)

Predicted Cases (log)

Predicted Cases (log)

**Figure S1. Comparing Predicted and Observed COVID-19 Cases (lagged 5 days) for Models A-F**

**B**. Quadratic Pre-Trend

**A**. Linear Pre-Trend

Predicted Cases (log)

Predicted Cases (log)

AIC=16.4

AIC= 16.4

AIC= 169.3

COVID-19 Cases (log)

COVID-19 Cases (log)

**F**. Poisson

Predicted Cases (log)

COVID-19 Cases (log)

**Notes:** All models predict cumulative daily COVID-19 cases (lagged 5 days) using a generalized linear model. Models A-C assume a negative binomial distibution with a log link. Models D-F assume negative binomial, gamma, and poisson distributions, respectively.

**AIC=Akaike Information Criterion.**

**Table S1.** State-specific estimates of COVID-19 cases averted with social distancing policies compared to projected cases without social distancing (three weeks after school closure)

|  | Difference in COVID-19 cases/100,000 residents | Difference in total cumulative COVID-19 cases |  |
| --- | --- | --- | --- |
| New York | -61,045 | -11,900,000 |  |
| New Jersey | -43,347 | -3,861,562 |  |
| Louisiana | -23,714 | -1,105,075 |  |
| Massachusetts | -19,759 | -1,363,805 |  |
| Connecticut | -19,582 | -699,609 |  |
| Michigan | -13,441 | -1,343,512 |  |
| Rhode Island | -11,980 | -126,665 |  |
| District of Columbia | -11,474 | -80,601 |  |
| Washington | -11,102 | -836,622 |  |
| Illinois | -10,131 | -1,290,798 |  |
| Delaware | -10,050 | -97,205 |  |
| Colorado | -9,854 | -561,239 |  |
| Indiana | -7,057 | -472,235 |  |
| Iowa | -6,703 | -211,565 |  |
| Vermont | -6,473 | -40,538 |  |
| Pennsylvania | -6,001 | -768,582 |  |
| Mississippi | -5,676 | -169,517 |  |
| California | -5,519 | -2,183,286 |  |
| Maryland | -5,515 | -333,273 |  |
| Idaho | -5,468 | -95,927 |  |
| Georgia | -4,817 | -506,675 |  |
| Nevada | -4,411 | -133,845 |  |
| New Hampshire | -4,312 | -58,485 |  |
| Tennessee | -3,959 | -268,033 |  |
| New Mexico | -3,956 | -82,903 |  |
| Wisconsin | -3,834 | -222,885 |  |
| Ohio | -3,804 | -444,717 |  |
| Utah | -3,711 | -117,297 |  |
| Florida | -3,303 | -703,459 |  |
| Alabama | -3,206 | -156,690 |  |
| Virginia | -3,084 | -262,649 |  |
| Maine | -2,864 | -38,336 |  |
| Missouri | -2,776 | -170,088 |  |
| Texas | -2,614 | -750,316 |  |
| Kansas | -2,577 | -75,018 |  |
| Oregon | -2,544 | -106,630 |  |
| Kentucky | -2,509 | -112,102 |  |
| Minnesota | -2,364 | -132,674 |  |
| South Carolina | -2,309 | -117,374 |  |
| West Virginia | -2,220 | -40,083 |  |
| Alaska | -2,135 | -15,744 |  |
| Montana | -2,104 | -22,354 |  |
| South Dakota | -2,094 | -18,474 |  |
| Hawaii | -2,021 | -28,706 |  |
| North Carolina | -1,993 | -206,931 |  |
| Oklahoma | -1,985 | -78,284 |  |
| Arizona | -1,970 | -141,288 |  |
| Nebraska | -1,875 | -36,173 |  |
| North Dakota | -1,512 | -11,490 |  |
| Arkansas | -1,478 | -44,531 |  |
| Wyoming | -1,405 | -8,115 |  |
|  |  |  |  |
| Total |  | -32,987,236 |  |

Notes: Cumulative daily cases were estimated using a generalized linear model with a negative binomial distribution and a log link and offset to account for state population. For each state, we used the method of recycled predictions to compare predicted cumulative cases with social distancing compared to without social distancing at three weeks after the date of school closure.
